# Concomitant Variations of the Scapular Region Musculature With an Aberrant Neurovascular Pattern

**DOI:** 10.7759/cureus.104929

**Published:** 2026-03-09

**Authors:** Sandeep Saluja, Sarika R Tigga, Sushil Kumar

**Affiliations:** 1 Anatomy, Amrita School of Medicine, Faridabad, IND; 2 Anatomy, University College of Medical Sciences & Guru Teg Bahadur Hospital, Delhi, IND

**Keywords:** axillary nerve, infraspinatus, latissimus dorsi, posterior circumflex humeral artery, rotator cuff, teres major

## Abstract

The shoulder complex exhibits a distinctive amalgamation of muscular power, integration, and an extensive range of motion. The shoulder joint allows for a wide range of upper limb movements because the four small muscles that make up the musculotendinous rotator cuff work together to keep the joint stable. The four muscles that come from the scapula and form the rotator cuff are the subscapularis, supraspinatus, infraspinatus, and teres minor. Accurate diagnosis and proper management of shoulder-related injuries are essential, particularly for sportspersons and individuals engaged in heavy weightlifting activities. The findings mentioned in this paper were observed during standard cadaveric dissection conducted for undergraduate medical students. Upon the methodical excision of skin and fascia in the scapular region, the findings detailed below were noticed and documented.

The infraspinatus muscle was observed to arise from the medial two-thirds of the infraspinous fossa, splitting into two parts, each inserting separately onto the greater tubercle of the humerus. The terminal tendon of the teres major was noticed to attach directly into the posterior and superior parts of the latissimus dorsi tendon. Simultaneously, an atypical vascular configuration was observed in the arm, in which the posterior circumflex humeral artery (PCHA) and the profunda brachii originated together from the brachial artery in the arm. The PCHA then travelled between the long and lateral heads of the triceps, situated within the lower triangular space. The artery ascended to go with the axillary nerve as it emerged through the quadrangular space. Finally, the PCHA supplied the deltoid muscle, marking its termination. Variations in shoulder girdle musculature, combined with an atypical vasculature, may aggravate shoulder injuries, particularly rotator cuff repairs, and enhance the technical complexity of surgical interventions.

## Introduction

The pectoral girdle is distinguished by its remarkable blend of muscular power, coordinated movement, and a wide range of mobility. This freedom results from the movements of the synovial multiaxial spheroidal glenohumeral joint formed between the hemispherical humeral head and the shallow glenoid fossa of the scapula. The joint stability is dependent on the integrity of four small muscles that merge with the joint capsule to make the musculo-tendinous rotator cuff (RC). These four muscles, which are referred to as the subscapularis (SSc), supraspinatus (SS), infraspinatus (IS) and teres minor (TMi), arise from the scapula and then attach onto the proximal humerus. The subscapularis inserts onto the lesser tubercle, while the remaining muscles insert onto three impressions of the greater tubercle of the humerus [1]. Lesions of the RC are associated with advancing age, trauma, limb dominance, smoking, hypercholesterolaemia, kyphotic-lordotic and flat-back posture and occupations demanding heavy labour [2]. Massive tears present an array of clinical symptoms. which may be treated by tendon transfer surgeries. RC repair frequently requires the transfer of the teres major (TMj) or latissimus dorsi (LD) tendons to the humeral head as they adduct and produce medial rotation of the humerus in conjunction, and also assist in the extension of the arm. Such procedures require utmost care owing to the proximity of the axillary (AxN) and radial nerves (RN) to the aforesaid structures [3]. The AxN (root value C5, C6) is seen in the axilla at the lower border of the SSc, where it winds posteriorly with the posterior circumflex humeral vessels through the quadrangular space (of Velpeau). This space is superiorly delineated by the TMi, inferiorly by the TMj, medially by the long head of the triceps, and laterally by the surgical neck of the humerus. The AxN bifurcates into anterior and posterior branches inside this quadrangular space. The anterior branch encircles the surgical neck of the humerus to supply the deltoid, whilst the posterior branch travels backwards to innervate the teres minor and the skin covering the lower two-thirds of the posterior deltoid as the lateral brachial cutaneous nerve [4].

Considering the importance of knowledge of normal and variant anatomy of the scapular region in surgical repairs of shoulder injuries, we report a unique case of the accessory IS muscle, conjoined tendons of TMj and LD with variant origin and course of the posterior circumflex humeral artery (PCHA). This article was previously presented as a conference abstract/poster at The National Anatomy Conference - "RRR" Anatomy: The Recent, Relevant, and Research-based, Army College of Medical Sciences (ACMS), Delhi Cantt. on 26th and 27th July 2024.

## Case presentation

**Figure 1:**
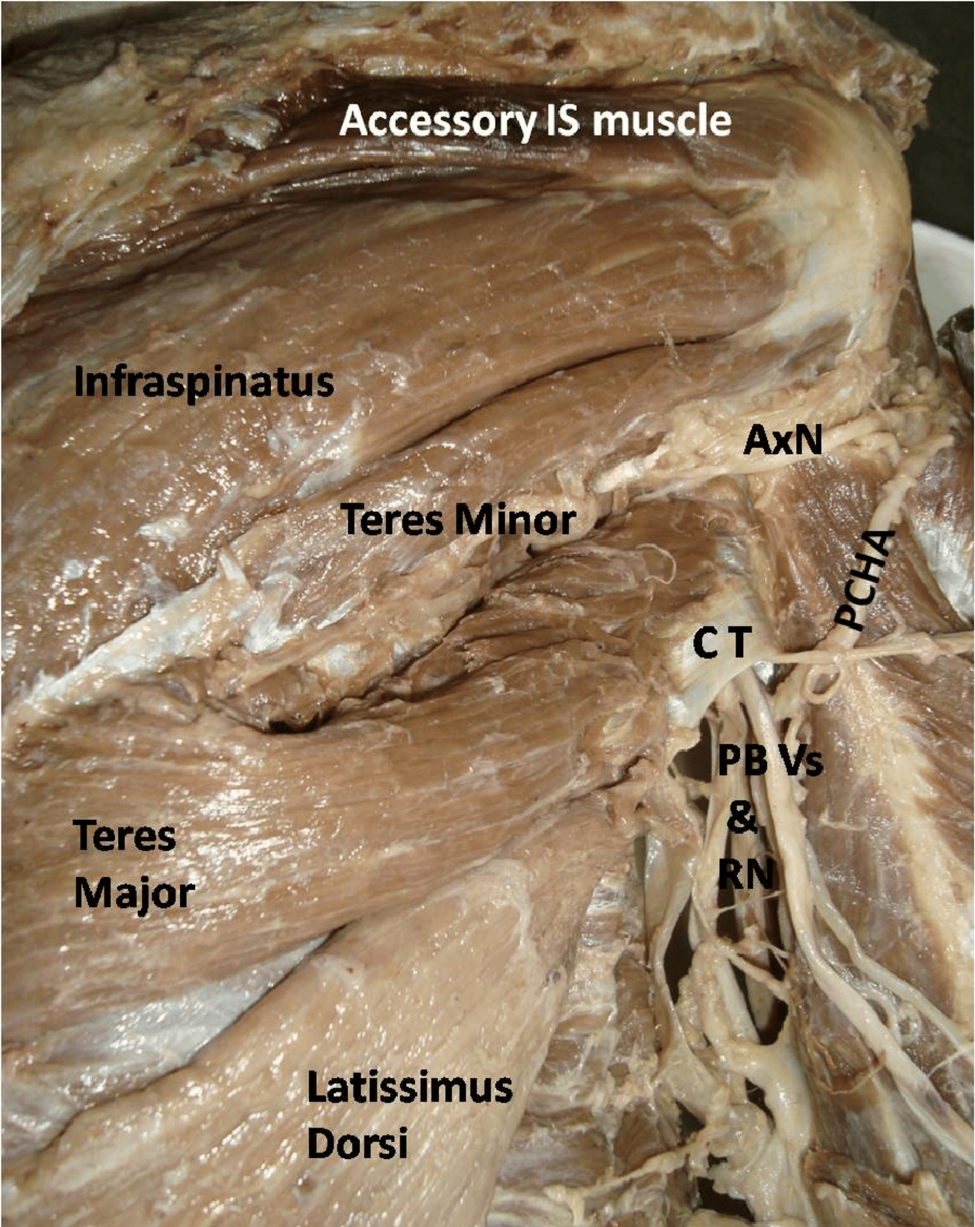
Dissected scapular region showing accessory infraspinatus (IS) muscle, infraspinatus, teres minor, teres major and latissimus dorsi CT: conjoint tendon of teres major and latissimus dorsi; AxN: axillary nerve; PCHA: posterior circumflex humeral artery; PB Vs: profunda brachii vessels; RN: radial nerve

A rare anatomical presentation of the right scapular region was observed during routine anatomical dissection of a 55-year-old male cadaver. Upon examination of the rotator cuff muscles, the IS muscle was observed to originate from the medial two-thirds of the infraspinous fossa and divide into two parts, each having a distinct insertion on the greater tubercle of the humerus. The upper, smaller part (accessory IS muscle) arose from the lower surface of the spine of the scapula and inserted onto an impression between the insertions of SS and the remaining IS (Figure 1).

**Figure 2:**
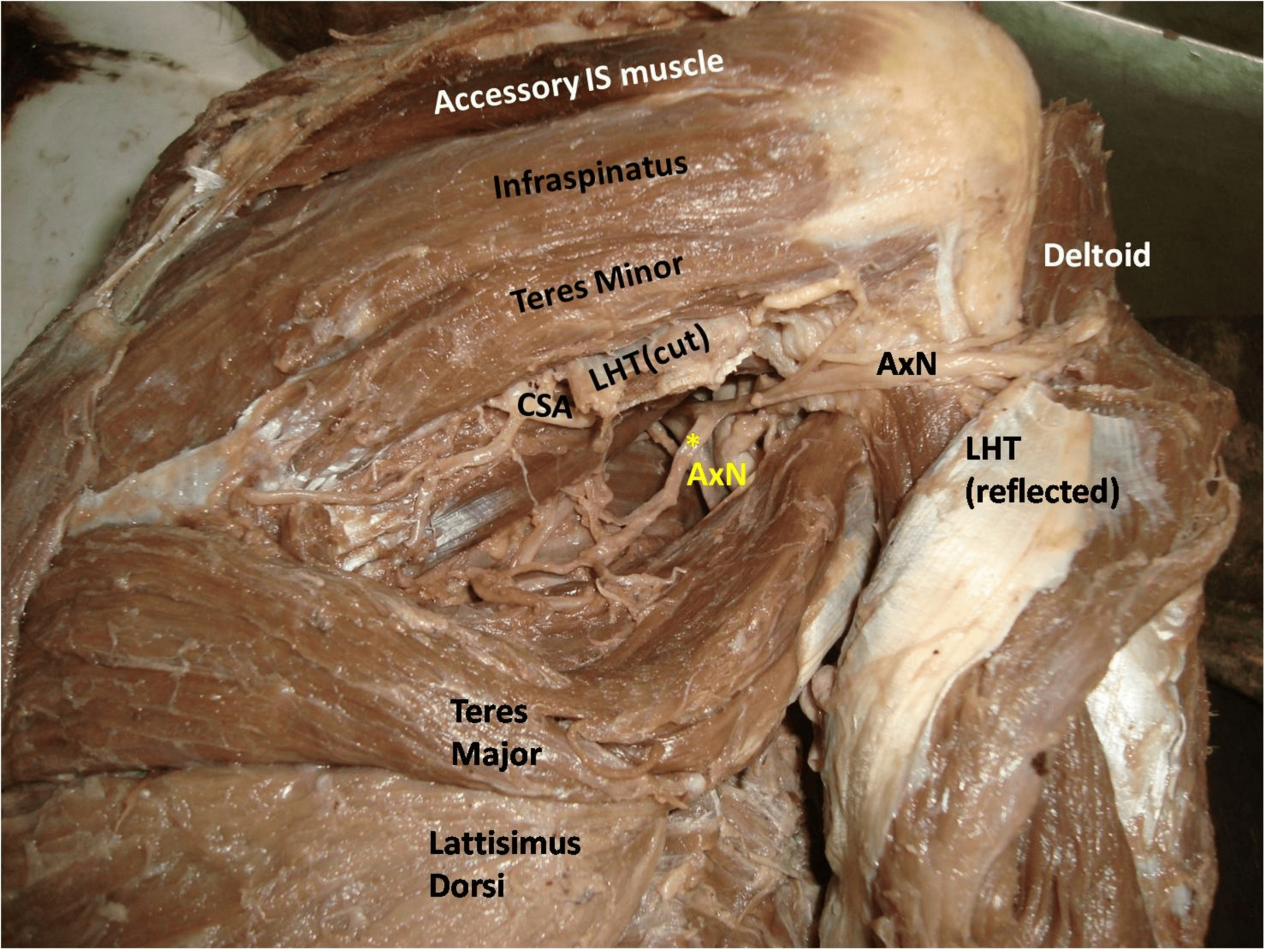
Dissected intermuscular spaces of the scapular region showing the axillary nerve supplying the teres minor and teres major muscles LHT: long head of triceps brachii muscle; AxN: axillary nerve; CSA: circumflex scapular artery

Both the bellies were supplied by the suprascapular nerve. Concurrently the muscle fibres of the TMj were found to insert onto the superior border and posterior surface of the LD tendon. Interestingly, the TMi and TMj muscles were both supplied by the AxN (Figure 2).

**Figure 3:**
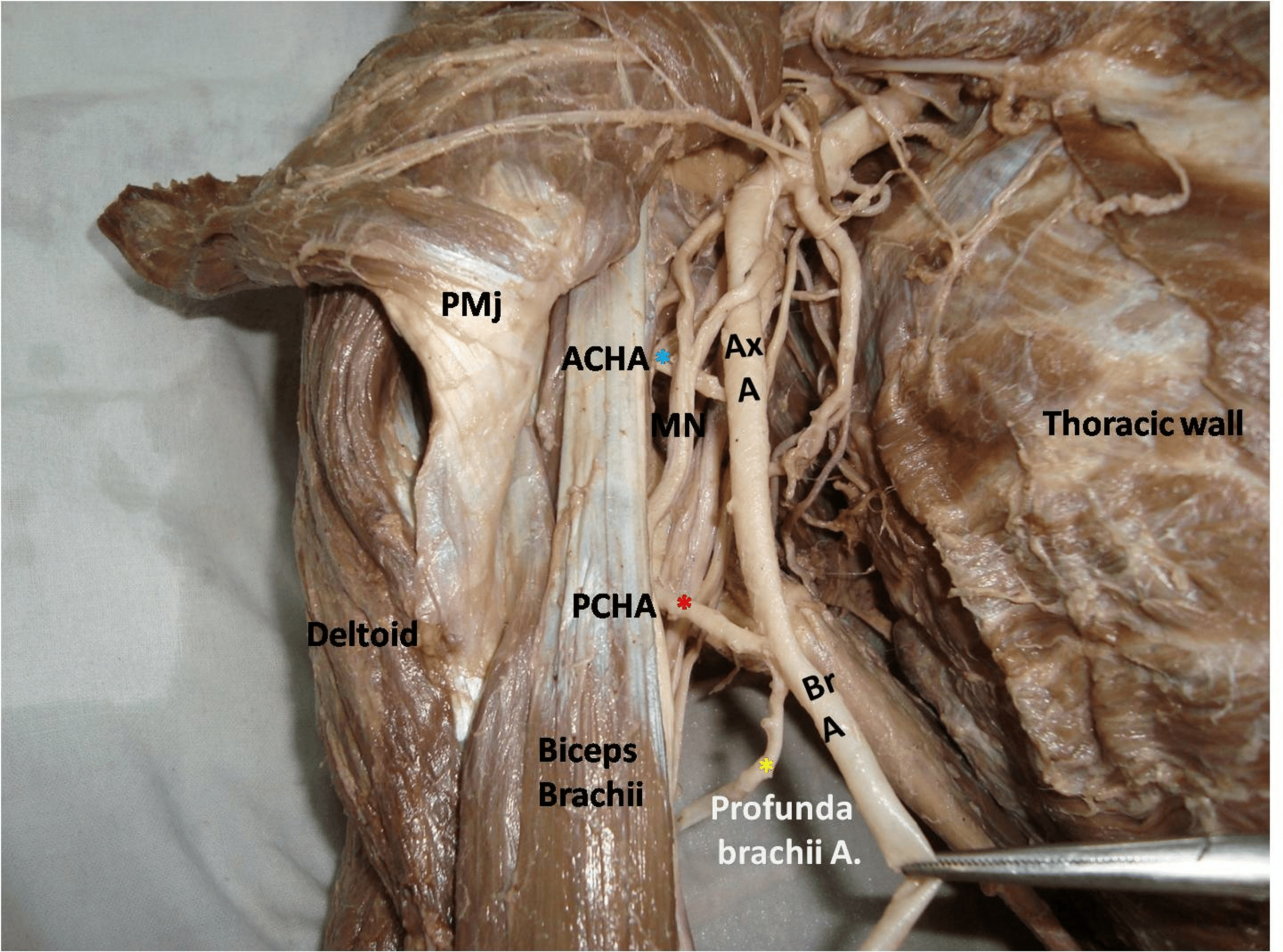
Dissected axillary region showing origin of posterior circumflex humeral artery from the brachial artery PCHA: posterior circumflex humeral artery (in red asterisk); Ax A: axillary artery; ACHA: anterior circumflex humeral artery (in blue asterisk); MN: median nerve; Profunda brachii A: profunda brachii artery (in yellow asterisk); Br A: brachial artery; PMj: pectoralis major

The axillary artery from its third part gave off the subscapular and anterior circumflex humeral arteries, while the PCHA arose from the brachial artery along with the profunda brachii (Figure 3).

**Figure 4:**
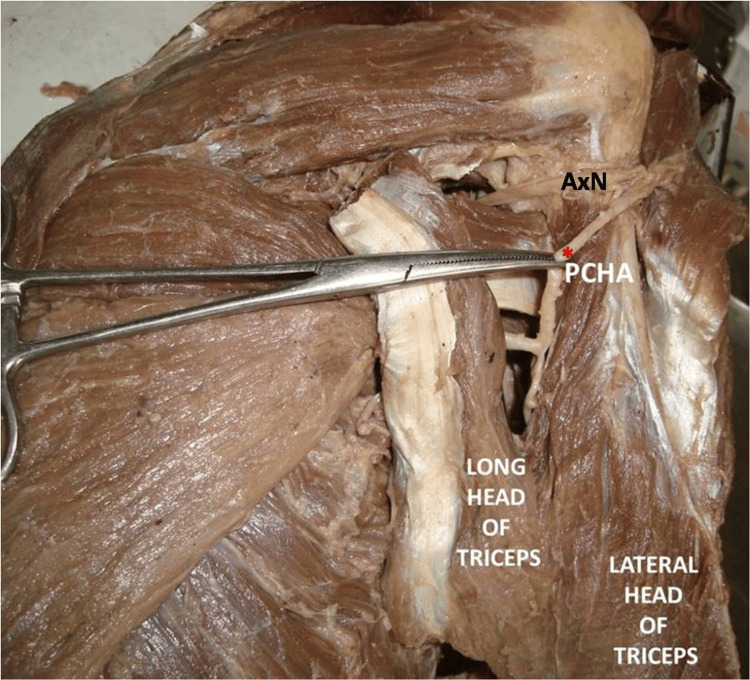
Dissected intermuscular spaces of the scapular region showing the posterior circumflex humeral artery traversing through the lower triangular space, then accompanying the axillary nerve PCHA: posterior circumflex humeral artery (in red asterisk); AxN: axillary nerve

**Figure 5:**
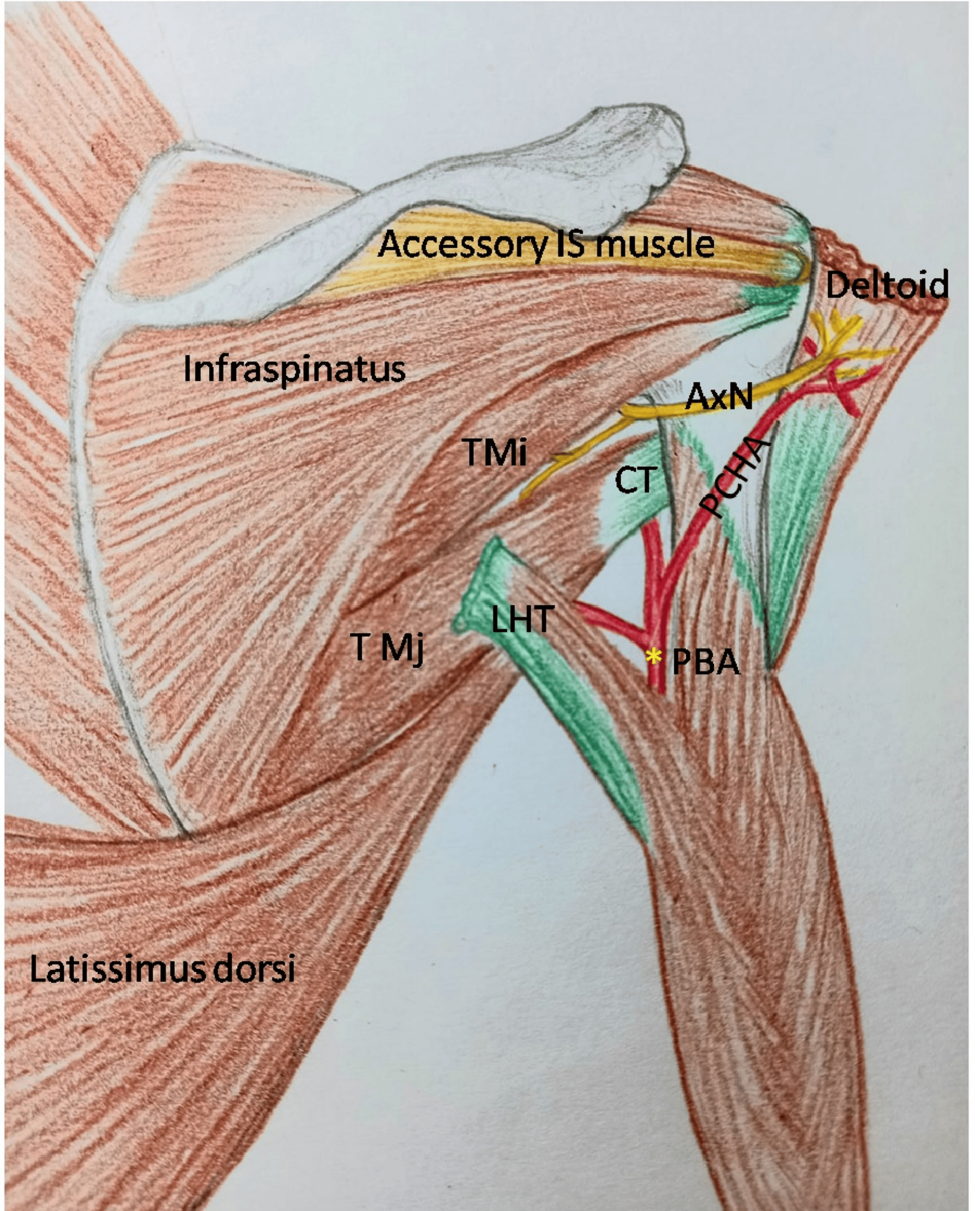
Diagram of the scapular region It shows the accessory infraspinatus muscle, the conjoint tendon of teres major and latissimus dorsi, the axillary nerve supplying the teres minor and teres major muscles, and the posterior circumflex humeral artery traversing through the lower triangular space and then accompanying the axillary nerve. IS: infraspinatus; CT: conjoint tendon of teres major and latissimus dorsi; TMi: teres minor; TMj: teres major; AxN: axillary nerve; PCHA: posterior circumflex humeral artery; PBA: profunda brachii artery (in yellow asterisk); LHT: long head of triceps. Image credit: Hand-drawn diagram created by the authors and labelled after transferring into Microsoft PowerPoint (Microsoft Corp., Redmond, WA, USA).

The PCHA was observed to travel backwards between the long and lateral heads of the triceps, located in the posterior aspect of the arm, as a component of the lower triangular space. Then the artery ascended to go along with the AxN as it emerged through the quadrangular space, subsequently supplying the deltoid muscle (Figures 4, 5).

## Discussion

Several researchers have reported variant anatomy of the muscles and vessels of the scapular region. Cobb et al. observed the accessory IS muscle bilaterally in an 83-year-old male cadaver. The anomalous muscle was thin and flat, composed of parallel fibres that arose from the medial dorsal surface of the scapula, passed beneath the scapular spine, and inserted onto the greater tubercle of the humerus, which was comparable to the present case [5]. Furthermore, Koptas et al. noticed that the IS muscle originated from the inferior aspect of the scapular spine and the infraspinous fossa and consisted of two distinct heads. The superior head was attached to the greater tubercle of the humerus, while the inferior head joined the tendinous portion of the infraspinatus. Additionally, an atypical fusion was observed between the IS and TMi muscles [6].

Among the most frequent diseases affecting the shoulder, RC tears are the most common. Most RC tears can be treated without surgery; some massive, irreparable cases necessitate tendon transfer surgeries. For a tendon to be suitable for transfer around the shoulder, it should permit adequate movement, and its relocation should result in minimal functional loss [3]. In tendon transfer surgeries for treating massive RC tears, the tendons of the pectoralis major, deltoid, trapezius, LD, and TMj muscles are commonly employed [7]. Studies have demonstrated that the LD muscle serves as an excellent pedicled flap due to its long neurovascular pedicle and substantial size [3]. Magermans et al. described that transferring the TMj tendon to the SS insertion yields excellent functional results in managing massive rotator cuff tears [8]. In an anatomical dissection of 12 cadavers, Goldberg et al. reported three insertional patterns of the LD tendon with respect to the TMj. In 67% of cases, completely separated LD and TMj tendons were observed; 25% of cases showed a loosely bound common tendinous insertion of both muscles; and in the third pattern (8%), the tendons were completely conjoined, as was seen in the present case [3]. Though in the present study, a transfer of the conjoined tendon of the LD and TMj can be performed, these combined tendons would demonstrate less excursion compared to the LD tendon alone. If the LD were to be transferred, the TMj would also need to be transferred along with it as a common musculotendinous unit.

The axillary artery (AxA), which continues from the subclavian artery, is divided into three segments by the pectoralis minor - one lying proximal to it, one posterior to it, and one distal to it. The AxA typically provides one branch from its first segment (the superior thoracic artery), two branches from its second segment (the thoracoacromial and lateral thoracic arteries), and three branches from its third segment (the subscapular, anterior circumflex humeral, and posterior circumflex humeral arteries) [1].

However, during routine dissections, variations of the axillary artery branching pattern are commonly observed, predominantly in the second and third parts [9,10]. Ongun et al. reported the presence of a common trunk arising just below the subscapular artery from the third part of the AxA, which gave rise to the anterior and posterior circumflex humeral arteries, the brachial artery, and the deep brachial artery [9]. Ramos-Alicea et al. described a variation in the branching pattern of the third part of the AxA, where the subscapular artery gave an atypical origin of the PCHA. After originating, the PCHA was accompanied by the AxN as it passed through the quadrangular space to supply the dorsal aspect of the shoulder joint and surrounding muscles [10]. In contrast, in the present case, the PCHA originated from the brachial artery along with the profunda brachii, passed through the lower triangular space, and then ascended to join the AxN, supplying the deltoid muscle.

The IS is composed of cross-striated muscle fibres that originate in the mesoderm. The muscles of the shoulder develop from an integrated embryonic muscle mass that fuses with the pectoral mass and the common arm sheath, collectively recognised as the upper limb bud. This bud is located opposite the somites C4, C5, C6, C7, C8, T1, and T2. By the fifth week of embryonic development, cells of the mesoderm originating from these somites migrate into the upper limb bud, where they differentiate into the anterior and posterior condensations. The posterior condensation subsequently forms the deltoid, SS, IS, TMi, TMj, SSc, anconeus, triceps brachii, brachioradialis, and the extensor muscles of the forearm [11].

When the embryo reaches about 11 mm in length, the developing scapular spine, along with the acromion, begins to divide the deltoid muscle mass horizontally, and the origin of the deltoid starts to form. During this period, the deltoid muscle mass also splits to form another mass that differentiates into the SS and IS muscles. This separation occurs between the 11-mm and 15-mm stages of embryonic growth. As development progresses, the IS gradually detaches from the TMi and extends to cover an increasing part of the scapular fossa, attaining full coverage when the embryo reaches approximately 20 mm in length [6,11]. Any deviations in this process may lead to the formation of accessory muscles, as seen in the present case.

The initial segment of the main trunk of the axis artery of the upper limb differentiates into the axillary and brachial arteries, whilst its distally located segment gives rise to the anterior interosseous artery. Disparities in the branching architecture of the axillary artery result from developmental defects in the vascular plexus of the upper limb bud. These discrepancies may arise from disruptions during different phases of artery development, leading to the regression, persistence, or redevelopment of certain channels, which ultimately results in variant origin and path of the key arteries of the upper limb [12].

## Conclusions

Knowledge of anatomical variations of the shoulder region, as observed in this rare combination of variant presentations, encompassing the accessory IS muscle, the conjoint tendon of TMj and LD, and the AxN supplying both the TMi and TMj muscles, is imperative in the surgical repairs of shoulder injuries and tendon transfer procedures. It minimises confusion and aids in optimal functional restoration. Furthermore, thorough awareness of the aberrant origin and course of PCHA may reduce angiographic misinterpretations and technical difficulties encountered during various surgical procedures involving the scapular region.
